# On the design and development of a handheld electrocardiogram device in a clinical setting

**DOI:** 10.3389/fdgth.2024.1403457

**Published:** 2024-08-09

**Authors:** Alejandra Zepeda-Echavarria, Niek C. M. Ratering Arntz, Albert H. Westra, Leonard J. van Schelven, Froukje E. Euwe, Herke Jan Noordmans, Melle Vessies, Rutger R. van de Leur, Rutger J. Hassink, Thierry X. Wildbergh, Rien van der Zee, Pieter A. Doevendans, René van Es, Joris E. N. Jaspers

**Affiliations:** ^1^ Department of Medical Technology and Clinical Physics, University Medical Center Utrecht, Utrecht University, Utrecht, Netherlands; ^2^ Department of Cardiology, University Medical Center Utrecht, Utrecht University, Utrecht, Netherlands; ^3^ Department of Cardiology, Meander Medical Center, Amersfoort, Netherlands; ^4^ Stichting Cardiovascular Biologie, Delft, Netherlands; ^5^ Netherlands Heart Institute, Utrecht, Netherlands; ^6^ Department of Cardiology, Central Military Hospital, Utrecht, Netherlands

**Keywords:** mobile ECG, clinical scientific research, wearables, design and development, TRL

## Abstract

Cardiovascular diseases (CVDs) are a global burden that requires attention. For the detection and diagnosis of CVDs, the 12-lead ECG is a key tool. With technological advancements, ECG devices are becoming smaller and available for home use. Most of these devices contain a limited number of leads and are aimed to detect atrial fibrillation (AF). To investigate whether a four-electrode arrangement could provide enough information to diagnose other CVDs, further research is necessary. At the University Medical Center Utrecht in a multidisciplinary team, we developed the miniECG, a four-electrode ECG handheld system for scientific research in clinical environments (TRL6). This paper describes the process followed during the development of the miniECG. From assembling a multidisciplinary team, which includes engineers, cardiologists, and clinical physicians to the contribution of team members in the design input, design, and testing for safety and functionality of the device. Finally, we detail how the development process was composed by iterative design steps based on user input and intended use evolution. The miniECG is a device compliant for scientific research with patients within Dutch Medical Centers. We believe that hospital-based development led to a streamlined process, which could be applied for the design and development of other technologies used for scientific research in clinical environments.

## 1 Introduction

According to the WHO (World Health Organization), cardiovascular diseases (CVDs) rank as the top global causes of mortality. Before 2019, CVDs accounted for 32% of deaths globally (1). Cases diagnosed with CVDs have increased to almost double numbers from 1990 to 2019, where it accounts today for approximately 523 million cases (2, 3). To diagnose CVDs, the primary diagnostic tool is the electrocardiogram (ECG), with the 12-lead ECG as the gold standard in medical centers. The ECG is a clinically validated tool and vastly used over the years (4). However, access to ECG devices is limited, especially outside medical facilities, leading to strain on healthcare systems and inconvenience for patients (5, 6). Reported costs for CVDs in the Dutch healthcare system are up to 6.8 billion euros and increasing over the years, 65% of these costs correspond to hospital care (7).

To address these challenges, the integration of home-based medical devices has gained traction, particularly in ECG monitoring (8, 9). The main benefit is that diagnosis can be made when the patient has complaints without going to a medical center (8, 10). For patients in high-risk situations, the use of home ECG devices could be essential as they could help to diagnose promptly in an acute situation (11). Diercks et al. showed that by using pre-hospital ECGs, ST-segment elevation myocardial infarction (STEMI) patients were re-perfused faster and showed a trend for lower adjusted risk of in-hospital mortality compared to patients with no pre-hospital ECG (12). Home ECG devices could also give reassurance to patients and avoid stressing the healthcare systems (13, 14).

There is a wide variety of ECG devices for home use on the market. The main characteristics of these devices are the limited number of leads (single to 3-lead), and length of recordings that could go from less than a minute to up to 30 days length (15–17). Even though there are many different ECG home devices, most of them are intended for the detection of rhythm disorders such as atrial fibrillation (AF) (17). For a few devices, their capabilities to detect other cardiac disorders have been explored but are limited in their outcomes due to position and number of leads provided during normal use (18, 19). Current devices might be limited, and there is a need for detection of other cardiac disorders than AF, further innovations are needed in ECG devices.

To develop a new ECG device, it should be properly designed and evaluated for use, ensuring they reach an appropriate Technology Readiness Level (TRL) before deployment (20). This is particularly crucial when developing medical devices, where safety, effectiveness and accuracy are prioritized. To ensure the performance and safety of medical devices clinical testing is key, but before this testing the device should be in a TRL5 to TRL7 development phase. By developing based on the TRL paradigm the distinct stages for development and clinical testing are milestones towards effective development (21, 22).

At the University Medical Center Utrecht (UMCU), in a multidisciplinary team we developed an ECG device with four dry electrodes, the miniECG. This device was developed to investigate whether we can obtain enough information to diagnose different cardiac disorders, from a device placed over the chest area with a limited number of electrodes. The miniECG was developed for scientific research in patients, and its research and developments could establish the foundation for a medical device.

In this paper, we provide a detailed description of the miniECG design and development process for scientific research in a clinical environment (TRL6). We describe the interactive collection of user requirements, co-design approach, design methodology, and process for the design of the miniECG for clinical research.

## 2 Methods

**Figure 1:**
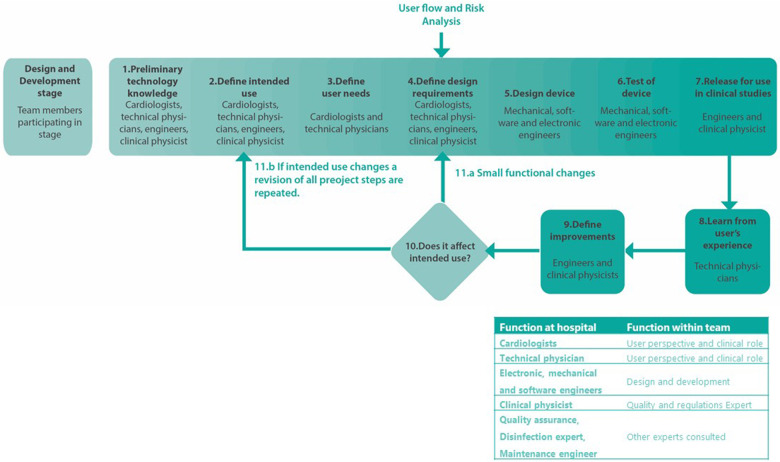
Development cycle for the design, development and release of the miniECG.

The development cycle of the miniECG for clinical research was possible by assembling a team combining different and complementary areas of expertise at the University Medical Center Utrecht (UMCU). Clinical physicists, cardiologists, and technical physicians provided initial inputs from users’ perspective, clinical considerations, and quality implications. The design and development activities were done by electronic, software, and mechanical engineers. If necessary other experts were consulted for design decisions (Figure 1).

Once the team was formed, key steps to develop a system suitable for use in clinical studies in Dutch Medical Centers were identified. Each step for the development is further detailed in the following sections (Figure 1).

### 2.1 Preliminary technology knowledge

Upon forming the multidisciplinary team, we performed a review of ECG devices to understand existing technologies. Our investigation involved examining both the 12-lead ECG, and ECG devices designed for home use. We were then able to understand their features, technological characteristics, and clinical evidence (17). From the experience of cardiologists, engineers, and technical physicians, we learnt about their previous efforts on a patented proof of principle that consisted of a handheld device with four position adjustable electrodes (23).

Drawing from the gold standard, ECG devices for home use, and the proof of principle, the team gained valuable insights guiding the design and development considerations for the miniECG, intended for scientific research in patients.

### 2.2 Define intended use

The intended use of the device was defined based on the expected use for scientific research in patients, while regarding the expected users and user scenarios (Table 1). This was later evaluated by the independent CE-mark committee within the University Medical Center Utrecht (UMCU). The committee concluded that the miniECG is not a medical device, due to the nature of the research which focuses on researching if sufficient information could be recorded to detect a broad variety of cardiac disorders. Another point was that no information for diagnosis was given to users so the device's use would not affect diagnosis and/or treatment decisions.

**Table 1:** Design and development cycle for versions 1.0 and 2.0. Movement sensor: accelerometer and gyroscopes sensors. Battery status: battery charge level.

Design and development stage		Output version 1.0	Output version 2.0
1	Intended use		
	User	Interventional and emergency nurses	Personnel acquiring 12-lead ECGs in the clinic
	Patient	Patients with chest pain	Patients visiting for check-up
	Research goal	Evaluate if miniECG could detect occlusive myocardial infarct by recording St-segment changes.	Evaluate if the miniECG could detect cardiac disorders via ECG and other sensors.
	Recordings	350 patients	>10 000 patients
2	User needs	• Easy to use • Multi-lead system • Dry electrodes • Smartphone size	• Easy to use • Multi-lead system • Dry electrodes • Smartphone size • Record cardiac activity with other sensors • User can see quality of recording
3	Design requirements		
	Housing	Size parameters, width (<8.0 cm), height (<12.0 cm), and thickness (<2.5 cm)	Size parameters, width (<8.0 cm), height (<12.0 cm), and thickness (<2.5 cm)
		Curvatures to accommodate anatomy of patients	Curvatures to accommodate anatomy of patients
			Modifications for visual feedback from device
			Water tightness
			Electronic board fixed to housing independently from electrodes
			Change material of device for robustness
	Electronic Board	Analog to digital converter to capture ECG	Analog to digital converter to capture ECG
		Microprocessor to process data and communicate with app	Microprocessor to process ECG, **movement sensor** data and communicate with app
			Lead-off detection visible on device
			Connection to electrodes and electronic board without mechanical restrain
			Improve Bluetooth communication
			Improve power consumption, device battery should last up to 300 recordings
	Electrodes	Biocompatible metal	Biocompatible metal
		With peaks to improve skin-electrode impedance	With peaks to improve skin-electrode impedance
	App	Record patient ID safely	Record patient ID safely
		Show lead-off detection on app	Show lead-off detection and **battery status** on app
		Trigger device to start recordings	Trigger device to start recordings
		Show steps for uploading data	Show steps for uploading data
			Received recorded data and show ECG for quality
4	Device Design	See Figure 6	
5	Test of Device	See Table 2	
6	Clinical release	Review TF and use device at emergency department and catheterization lab	Review TF and use device at heart function and out-patient clinic

### 2.3 Define user needs

To initiate the design process, we defined expected output based on research characteristics, users’, and patients’ perspectives. Each input defined characteristics to be complied with in the design of the miniECG system.

For the research, we considered input such as the data to be recorded and delivered. In terms of the users’ perspective, the device was to be operated by trained medical personnel and needed to consider how to make it easy to use. Finally, from patients’ perspective the device should comply with characteristics that allow to place and record ECGs without creating an inconvenience to patients.

All user needs were initially based on conversations with cardiologists and medical technicians, as they were users of the device and were in contact with the teams gathering the data at medical centers. They also had experience performing 12-lead ECGs and were in contact with patient candidates for the clinical studies.

### 2.4 Define design requirements

The design requirements were defined based on users’ needs, analysis of the users’ flow (Supplementary 1) and risk analysis activities. By reflecting on the users’ flow, we determined the design requirements regarding the definition of the communication protocol, information to show, and to ask to user (Supplementary 2).

To consider the safety of users, patients, and research, we followed the risk analysis procedure to identify hazardous situations. To reduce the risks, we determined additional safety requirements and necessary design and manufacturing steps to mitigate hazardous situations. We performed risk analysis within the multidisciplinary team to identify hazardous situations related to the design and use of the miniECG, and performed it based on medical device standards (24).

### 2.5 Design device

**Figure 2:**
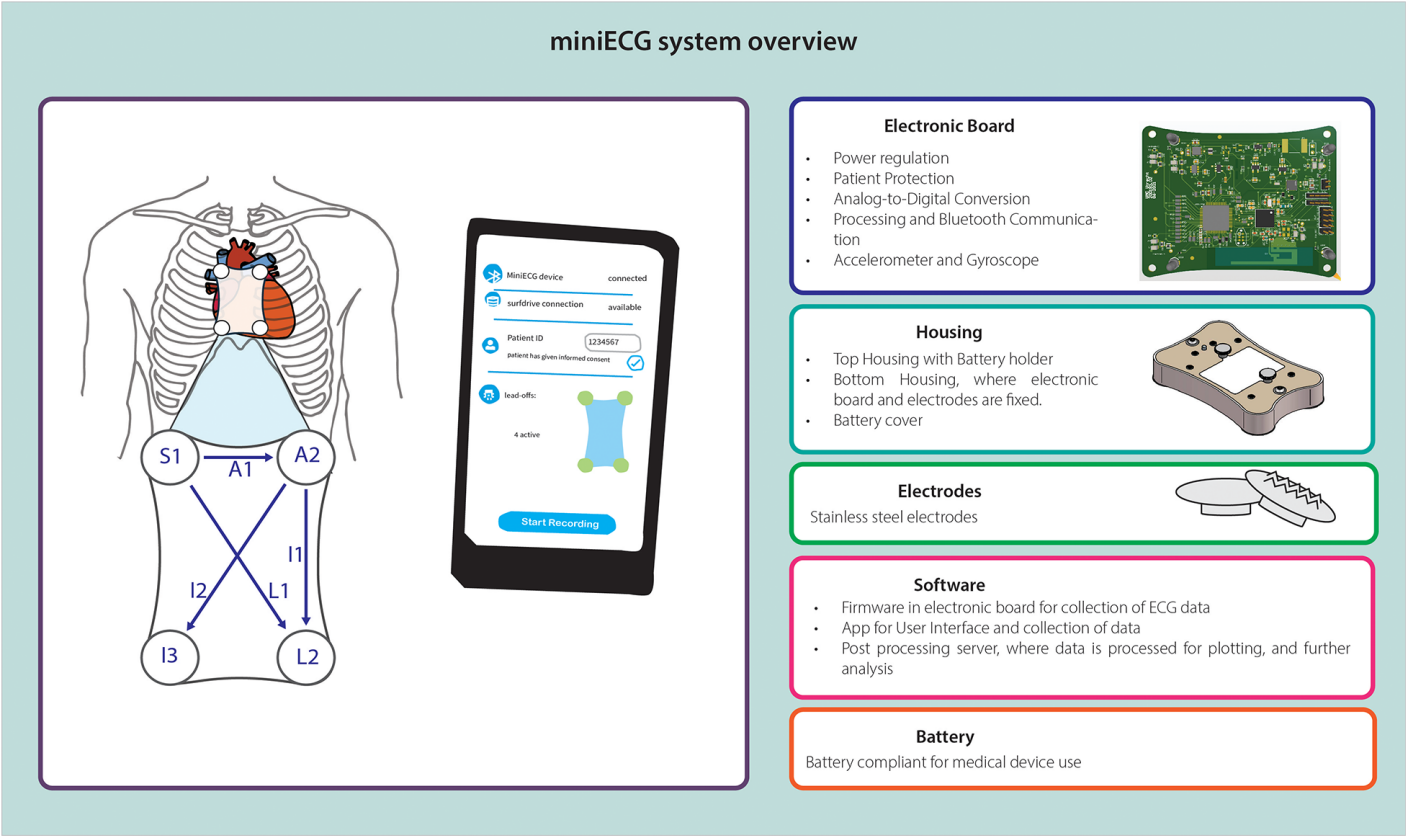
Overview of miniECG system with subsystem components and/or characteristics.

Based on the main characteristics and the risk analysis performed, we identified subsystems of the miniECG necessary to comply with the intended use and satisfy the user needs (Figure 2). Once the miniECG subsystems were designed and reviewed, they were integrated into the device.

### 2.6 Test of device

Testing activities were performed to evaluate the design, safety, and functionality of the system. Regarding safety, evaluations included electromagnetic interference from the miniECG to surrounding devices, and measurement of patient protection against leakage current to ensure electrical safety as per medical devices standards (25, 26). To evaluate the functionality of the system, tests comprised the evaluation of firmware and communication protocols. Finally, the product verification involved testing the electronic board, and assessing the accuracy and quality of recorded synthetic generated ECG signals (Table 2).

**Table 2:** Test performed on device for release.

Test Type	Test	Samples	Version 1.0	Version 2.0
Design verification	Mechanical design review	Design files	X	X
	Electronic design review	Design files	X	X
Safety testing	Pre-compliance EMC testing	1	X	X
	Leakage current	All manufactured devices	X	X
Functional testing	System interaction	1		
	Normal use cycle		X	X
	Errors		X	X
	ECG signal visible		/	X
	Battery state visible		/	X
	Automatic shutdown		/	X
Product verification	PCB (printed circuit board) verification	All manufactured devices		
	Voltage supplies		X	X
	LEDs		X	X
	ADC and simulated ECG		X	X
	Calibration	All manufactured devices		
	ECG calibration		X	X
	IMU calibration		/	X

X: Test performed on device. /: Test not applicable to device version.

### 2.7 Release of device

After testing and formal approval for use in scientific research. The miniECG system was ready for use at the UMCU.

### 2.8 Iterative design process

As previously mentioned, one of the first steps in the development of the miniECG was the definition of the intended use. But as research and uses of the device evolved, the intended use also changed (Table 1). Besides the uses of the miniECG based on the user's experience and risk analysis, improvements on the design of the device became necessary. Iterations on design were performed to comply with the evolution of the intended use and the improvements to guarantee functionality of the system. After every design cycle, the necessary documentation of the device was updated and if necessary, tests were added to evaluate the sub-systems and system, all of this was performed according to the quality system standards in the UMCU.

### 2.9 Quality compliance in a medical center

At the UMCU, it is possible to develop devices for use in the hospital. The development of such devices is done at the medical technologies and clinical physics department (MTKF) and does not require involvement of a Notified Body. The MTKF has a quality management system designed for the development of medical devices and has been certified as per ISO 13485.

Even though the miniECG has been designed as a research device only for scientific clinical research (TRL6), the design and development of the device followed departmental practices and procedures for development of medical devices. Together with clinical physicists and quality engineers we agreed on relevant documentation to prepare for the release of the devices for use in scientific research studies. A technical file (TF) of the device was compiled. Relevant information such as design specifications, risk analysis, technical drawings, source codes, test protocols, test reports among other files were created and gathered in the TF. The approval process for releasing the devices followed an established workflow approved by the legal affairs and board of directors of UMCU.

## 3 Results

**Figure 3:**
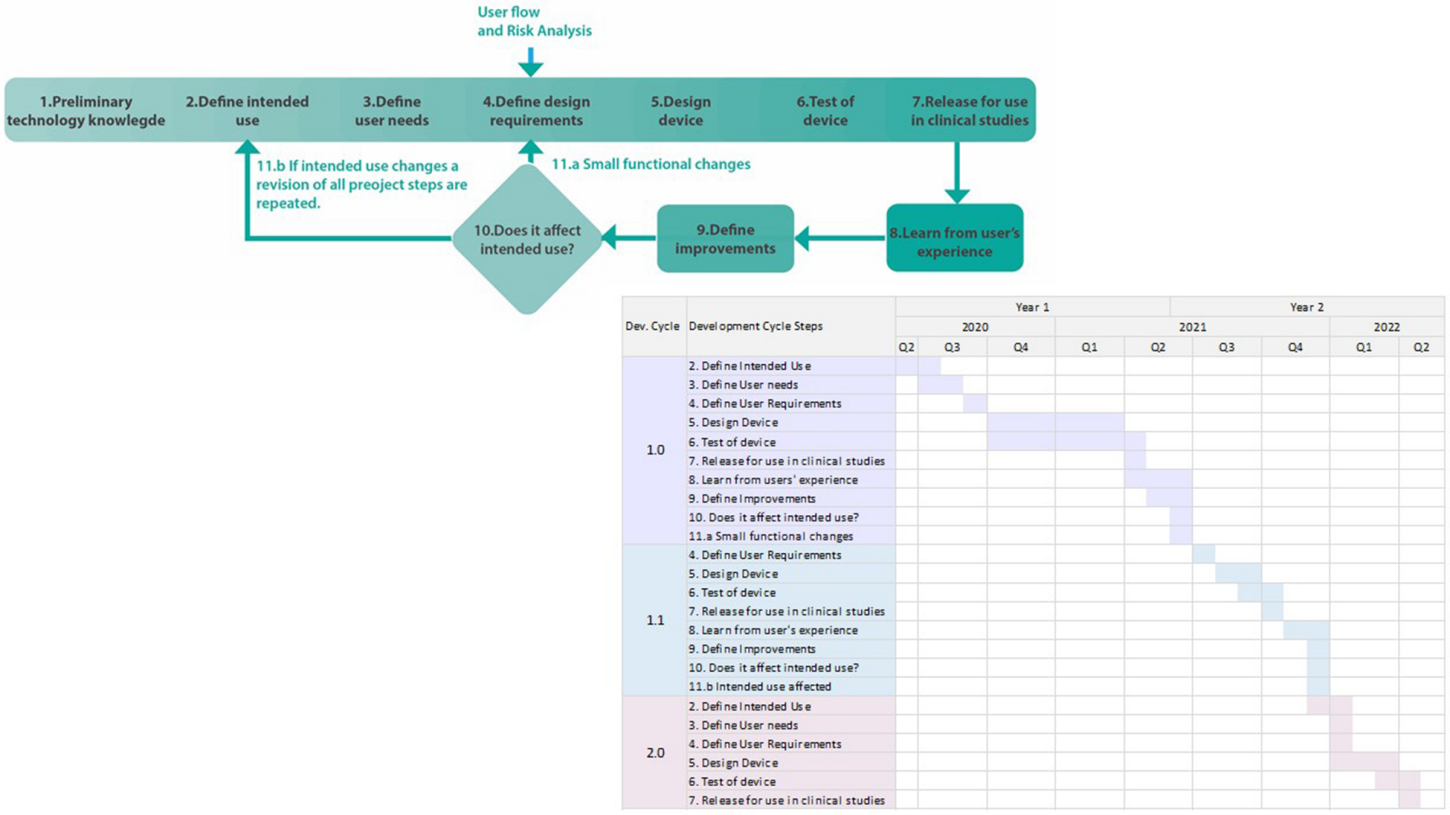
Timeline of development of miniECG with development cycle.

From June 2020 to May 2022 the development of the miniECG system was performed (Figure 3). The miniECG system in its first cycle (version 1.0) was designed and developed from June 2020 to April 2021, and it was released for scientific research designed to evaluate the capabilities of recording ECG changes in the presence of occlusive myocardial infarction (27, 28). As studies were running, feedback from users was collected by technical physicians and two iteration cycles were performed from May 2021 to April 2022. The second cycle (version 1.1) was performed for small improvements based on technical and manufacturing improvements. While for the third cycle (version 2.0) the main driver was to improve the users’ experience, quality of data and changes in the intended use (See Table 1).

The efforts on the development of the miniECG were possible due to the multidisciplinary team. As collaboration from the distinct expertise areas allowed for faster developments on the miniECG while considering technical, clinical, and quality perspectives. The development process was iterative, featuring evaluations before and after system integration, leading to continuous refinement of design, functionality, and safety features.

### 3.1 Development cycle and technology readiness

The development of the miniECG system was initiated to perform scientific research on patients. Research aimed to evaluate if sufficient cardiac information can be acquired for the detection of cardiac disorders by the system. In its first cycle (version 1.0) the goal was to evaluate the system for the detection of occlusive myocardial infarction, and third cycle (version 2.0) was aimed for the detection of cardiac disorders and to perform a broader comparison to the 12-lead ECG (Table 2).

**Figure 4:**
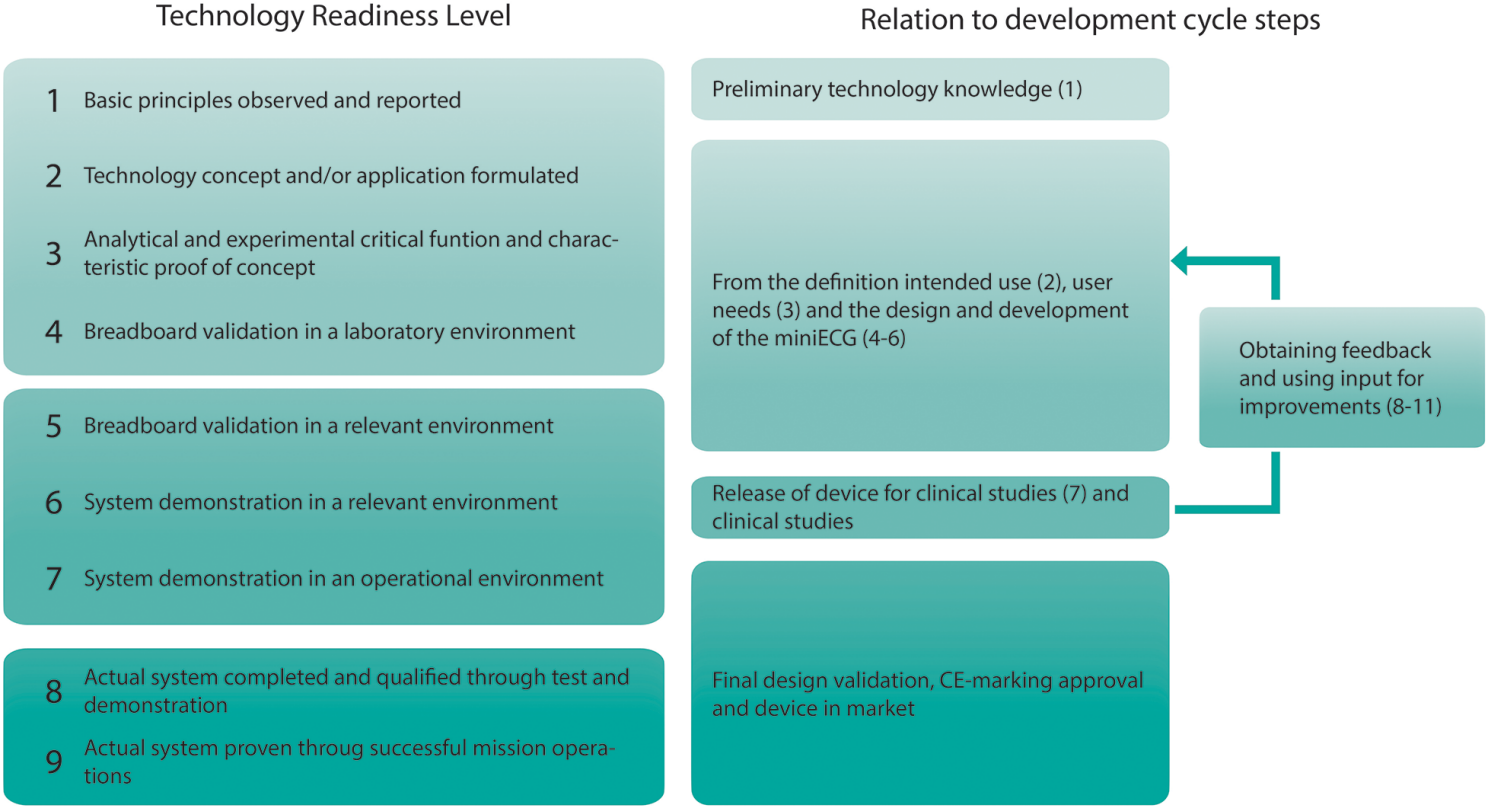
Technology readiness levels and the relation to the development cycle of the miniECG.

Research was the main driver for the development of the system. The development of the miniECG was to deliver systems for scientific research in clinical environments. When the development process of the system is compared to the technology readiness levels (TRL), we could see that every process step falls into different TRL levels, from the definition (TRL2), design (TRL3), and test (TRL4-5), and the use of the system for scientific research falls in TRL6 (Figure 4). Once improvements were necessary the system goes back to TRL2, and the process is reinitiated. Finally, as the system's end goal is for use in scientific research, further TRL steps are not necessarily fulfilled.

### 3.2 miniECG iterative process design

**Figure 5:**
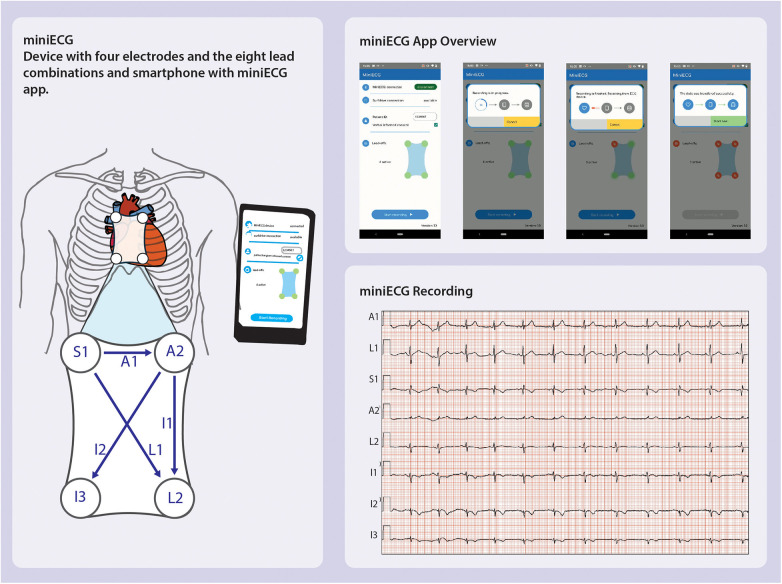
miniECG version 1.0 overview showing the leads recorded, app used in studies and resulting ECG recording (25, 26).

**Figure 6:**
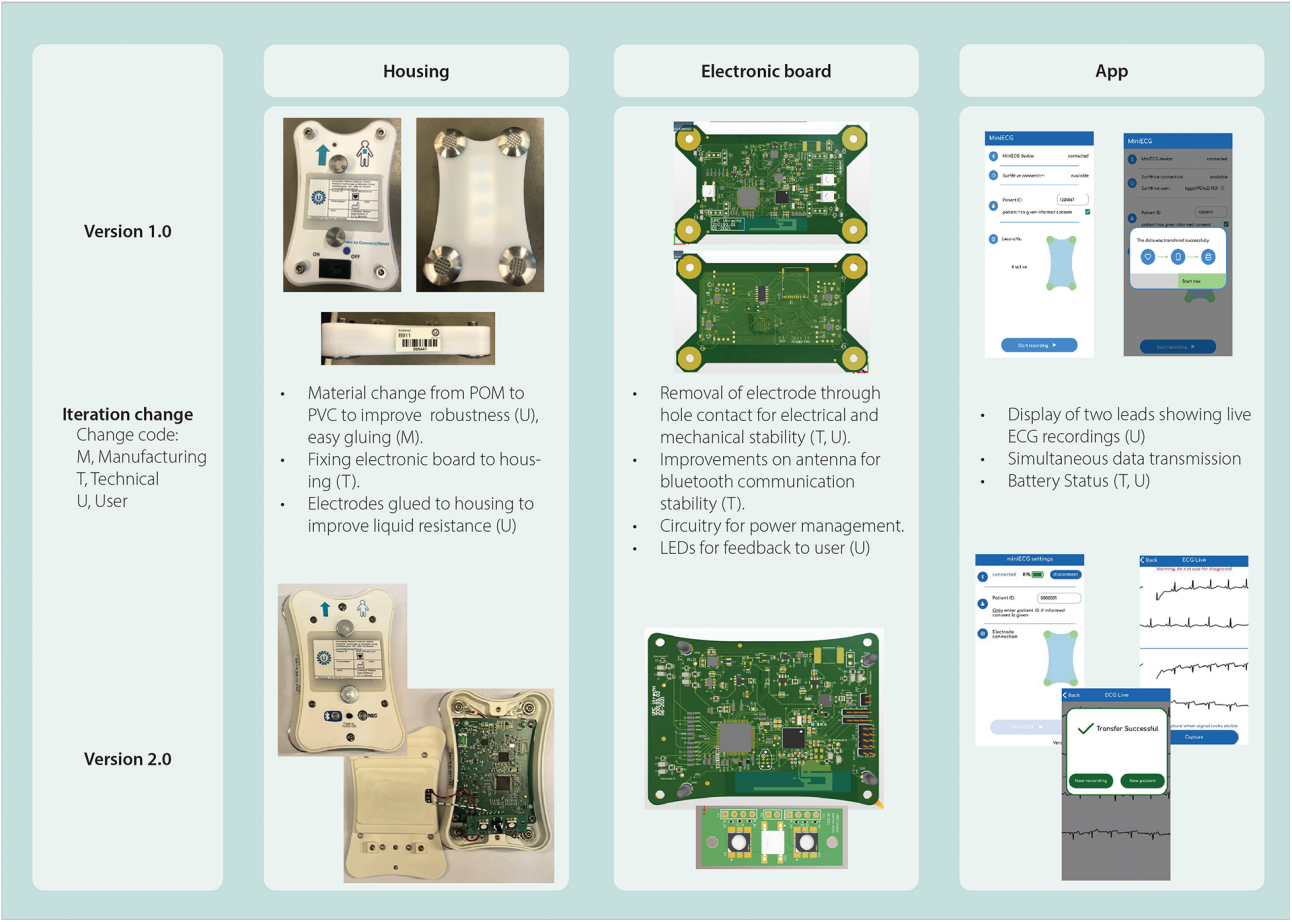
Overview of miniECG changes specifying origin of change, intended use and sub-systems changed.

The design of the miniECG started from the need to investigate if a system with a four-electrode arrangement could record ECGs in the chest area (Figure 5). As research progressed, it was evident that the miniECG needed improvements especially if more intense scientific research was to be performed (Figure 6). To develop the miniECG at the University Medical Center Utrecht, we developed the system based on the design cycle steps (Figure 1).

For the first and third design cycles (version 1.0 and 2.0, respectively) the intended use was one of the main drivers to the design (Table 1). Once version 1.0 was in use for studies, technical physicians were in contact with the users at the Medical Centers. They identified occasions when the system failed and communicated with the engineers about the issues. Issues reported ranged from the quality of recordings [∼24% inferior quality recordings (Supplementary 3)] to communication problems with the app causing loss of data (27, 28). Once engineers were aware of the issues, root cause analyses were performed. Improvements for the system were designed and implemented in further design cycles (Figure 6).

Open communication with the team was key for rapid identification of issues, technical physicians were in contact with users and engineers on a day-to-day basis, while the whole team would meet on a bi-weekly basis to follow-up progress of studies and development of the miniECG.

## 4 Discussion

In this study we explained the design and development process for the miniECG, a handheld device capable of recording multi-lead ECGs from a four-electrode arrangement on the chest area. The development of a safe, legally compliant, user friendly, and reliable device for use in scientific research studies in Dutch Medical Centers was possible due to multidisciplinary collaboration. Our comprehensive approach allowed us to develop a device where technical, quality, regulation and users’ perspectives were considered and integrated.

The development of the miniECG has been driven by the research goals and the multidisciplinary team. In its essence, the system is intended to investigate whether sufficient cardiac information is captured to detect cardiac abnormalities by the four-electrode arrangement contained in the miniECG. This approach might differ from startups developing medical devices, where the goal is to place a device in the market. The device developed by startups would need to demonstrate its safety, reliability, and accuracy. Kaplan et al. pointed out the pathway from prototype to initial clinical testing could take from 3 to 5 years (29). In comparison to the miniECG development where first design cycle took 1 year of development for first scientific research studies. As the miniECG has been developed for research, it is a distinct perspective than the intention to develop a medical device.

One of the characteristics of the development process was the multidisciplinary team within the University Medical Center Utrecht (UMCU). The department of medical technologies and clinical physics (MTKF) at the UMCU is certified to develop medical devices within the hospital by complying with the ISO 13485 (30, 31). We believe the reason we have been able to perform studies and improve the miniECG rapidly is because it has been developed in a special ecosystem where engineers, clinical and quality experts, and users were reachable in an efficient manner, as well as users and patients. As the development was in-house at the UMCU. Unlike companies where this will not be facilitated so easily.

For the design and development of the miniECG, we used an iterative design approach. On the first version of the system released, the development goal was to have a working version for the studies. This development approach allowed us to reduce the number of uncertainties while keeping a basic design for the miniECG. Follow-up version changes were then based on user experience, correction of design errors, and improvements for assembly. Other research groups have reported that similar approaches where combined Agile and V-model practices allowed to successfully develop medical embedded devices compliant with Medical Device Regulation (32, 33). Our approach was similar to Agile development. We had development cycles, goals and iterations which evolved with the development of the system, all possible by keeping open communication between team members and valuing their input on development stages.

One example of this was the design of the housing, while clinical roles mentioned that the device should be handheld and easy to clean, developers translated this to define maximum sizes in the three planes and review the material for the housing for cleanliness. The first iterations of the housing were 3D printed which allowed to see the fitting of components, and an initial plan was to have a 3D printed version. But upon consulting with hygiene experts, they recommended changing to a smooth material as the grooves generated during the extrusion of 3D printer material could trap dirt and would not be easy to clean. We then chose to use Polyoxymethylene (POM). As the studies progressed, the quality of ECGs was poor (Supplementary 3). Upon our analysis, we concluded that the housing needed to be more robust, and electrodes and electronic board needed to be fixed separately to the housing, so no mechanical constraints affect the quality of the ECGs. These challenges led to the change of material from POM to polyvinyl chloride (PVC).

Even though the miniECG is ready for use in scientific research studies, there are limitations on its current implementation. If the device is to be used as a medical device and the trajectory was set to place it in the market, further challenges are foreseen. The challenges start from developing for manufacturability, further testing and regulatory approval would require further efforts and investment (21, 22, 29). These challenges are present in further TRL stages outside the development scope at the UMC Utrecht.

The miniECG has proven to be a tool that could be used rule in the occlusion of coronary arteries, initially the animal study demonstrated that the miniECG can record ST-segment changes in a porcine model (28). And in our first pilot study, we demonstrated that the miniECG captures myocardial obstructions in multiple locations (27). Future design cycles are already in progress and conceptual testing is being performed. The option to add other complementary sensors is being evaluated. The idea for including other sensors is based on the concept that mechanical function of the heart could be recorded as well so that more information of the patient's cardiac activity is obtained (34, 35). As the miniECG only contains four electrodes for the measurement of ECGs, we believe that to add to its diagnostic value other sources of information, namely sensors could add complimentary information on the heart's state.

As different disciplines work together on the design and improvement cycles of a new device, as well as potentially new inventions (Intellectual property), it is valuable to keep records during development stages (36). We believe it is important to keep records in engineering notebooks or similar reports detailing which members were involved in potential inventive steps, to avoid discussions about inventorship afterwards.

As we prepare for future studies, we have involved other key members such as heads of department in the outpatient clinic, and patient groups. We would like to investigate if the use of the miniECG provides benefits while following up patients with chronic heart diseases.

On the development of medical devices in clinical environments, the methodology in this article could provide guidance on the implementation of new technologies. To move forward in the development of medical devices, we believe is necessary to have processes that allow change and evolution of devices. For that, the transparency of processes and designs would allow to new developments. In this manuscript, we aimed to show our development process. For future discussions on the miniECG design, we expect to use guidelines on digital health implementations to further on describe our design and design choices (37).

The monitoring of health of people has become of more importance, as well as the need to relieve the current care system from its high demand. The use of new technologies at homes such as PPGs, accelerometers, gyroscopes, and doppler radar technologies for the detection of vital signs has become crucial and necessary (35, 38–40). With these new developments to bring them for use at home, we invite researchers to follow our process for dynamic development of medical devices and clinical testing.

## 5 Conclusion

We developed the miniECG, a handheld device capable of recording ECGs via a four-electrode arrangement within a clinical environment. The success in developing the device is due to the multidisciplinary collaboration and iterative stages in its design. With the inclusion of distinct roles of the team we developed a reliable, user-centric device ready for use in scientific studies in Dutch Medical Centers.

## Data Availability

The raw data supporting the conclusions of this article will be made available by the authors, without undue reservation.
