# Supplementary material for: On the design and development of a handheld electrocardiogram device in a clinical setting
Source: Front Digit Health. 2024 Aug 9;6:1403457. doi: 10.3389/fdgth.2024.1403457 (PMC11341539; doi:10.3389/fdgth.2024.1403457)
Supplement: Supplementary file 1 [file Datasheet1.docx]

Supplementary Material 1
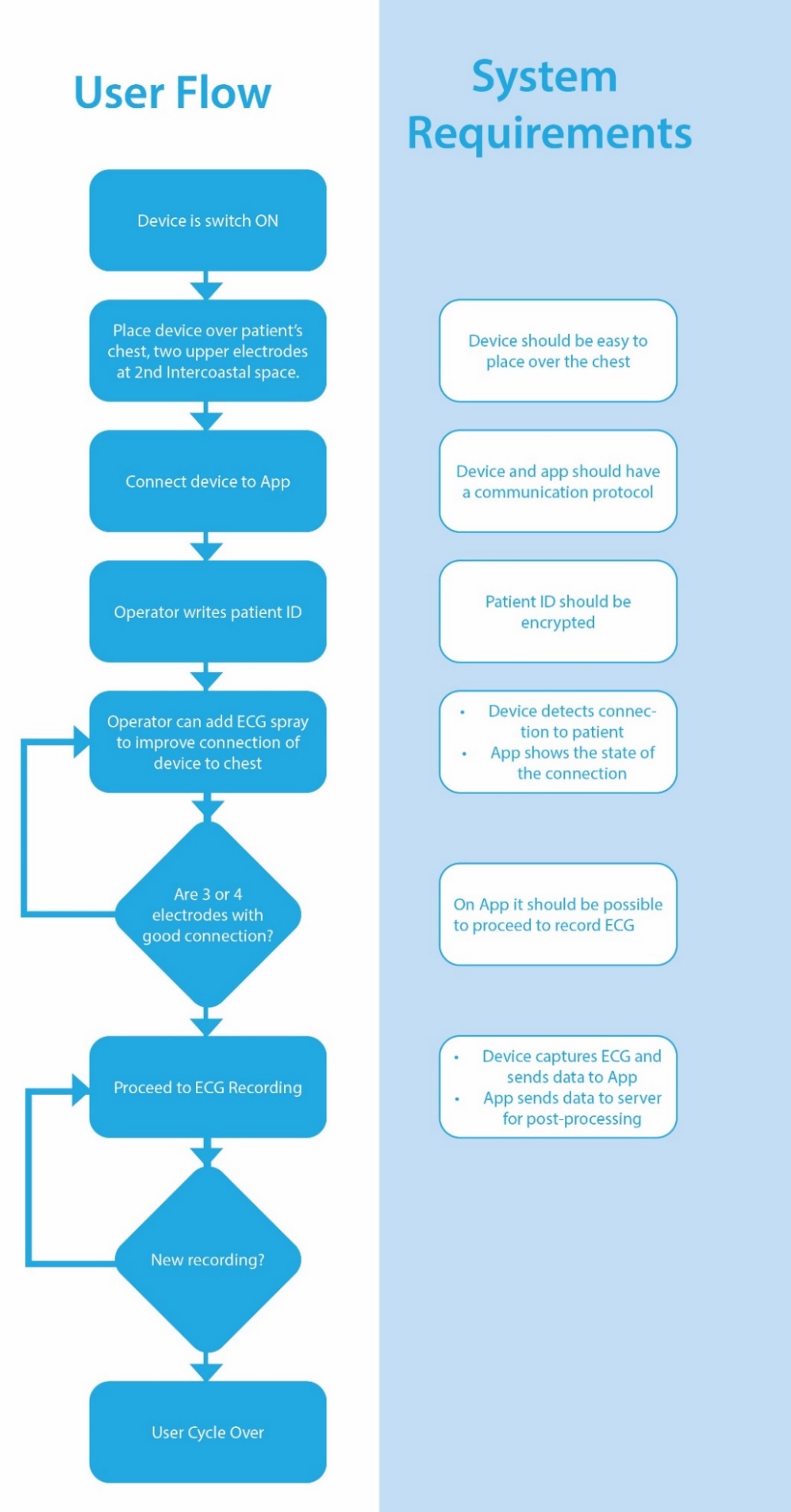


**Supplementary Figure 1.** Expected user flow during normal use of miniECG and system requirements that device should comply with to follow flow.
