# Supplementary material for: On the design and development of a handheld electrocardiogram device in a clinical setting
Source: Front Digit Health. 2024 Aug 9;6:1403457. doi: 10.3389/fdgth.2024.1403457 (PMC11341539; doi:10.3389/fdgth.2024.1403457)
Supplement: Supplementary file 2 [file Datasheet2.docx]

Supplementary Material 2

**Supplementary 2 Table 1**. Design characteristics of miniECG

| **Sub-system** | **Design Input** | **Design decision** | **Origin of design decision** |
| --- | --- | --- | --- |
| **Electrodes** | Reusable | Dry electrodes that can be used more than one time | User |
|  | Good contact with patient | Low impedance measurements | Technical |
|  | Do not irritate patients' skin | Material of electrodes should be biocompatible | Safety |
| **Electronic board** | Patient Protection | Patient protection for connection points | Safety |
|  |  | Power supply protection | Safety |
|  | Capable to record data from four electrodes | Signal instrumentation to record ECG | Technical |
|  |  | Data Processing and being able to record ECG | Technical |
|  | Capable to connect to smartphone | Transfer of recorded data to an app | Technical |
| **Housing** | Capable to fit PCB (Printed Circuit Board) and electrodes | Design to find PCB and Electrodes, as well as keeping a good connection within components | Technical/User |
|  | Design a handheld device | Size of device cannot be bigger than a smartphone, either for future use to be integrated in a cover or to keep it as a mobile device. | User |
|  | Easy to clean | Material selection should allow to easily clean the device | Safety |
|  | Female and Male users can use the system | Mechanical restrictions due to anatomical characteristics (e.g., device should lay on chest) of patients should be accounted for as much as possible. | User |
| **App** | Possible to record ECG | On app data such as data and pseudonymized patient Id should be saved as part of collected data | Technical/ safety |
|  |  | App should trigger device to start recording | Technical |
|  |  | App should collect and save data | Technical |
|  | User should be able to interact with app | User can add comments to recording | User |
|  |  | User can start and stop recordings | Technical |
|  |  | User can see recording steps on App | User |
