# Supplementary material for: On the design and development of a handheld electrocardiogram device in a clinical setting
Source: Front Digit Health. 2024 Aug 9;6:1403457. doi: 10.3389/fdgth.2024.1403457 (PMC11341539; doi:10.3389/fdgth.2024.1403457)
Supplement: Supplementary file 3 [file Datasheet3.docx]

Supplementary Material 3: ECG recordings


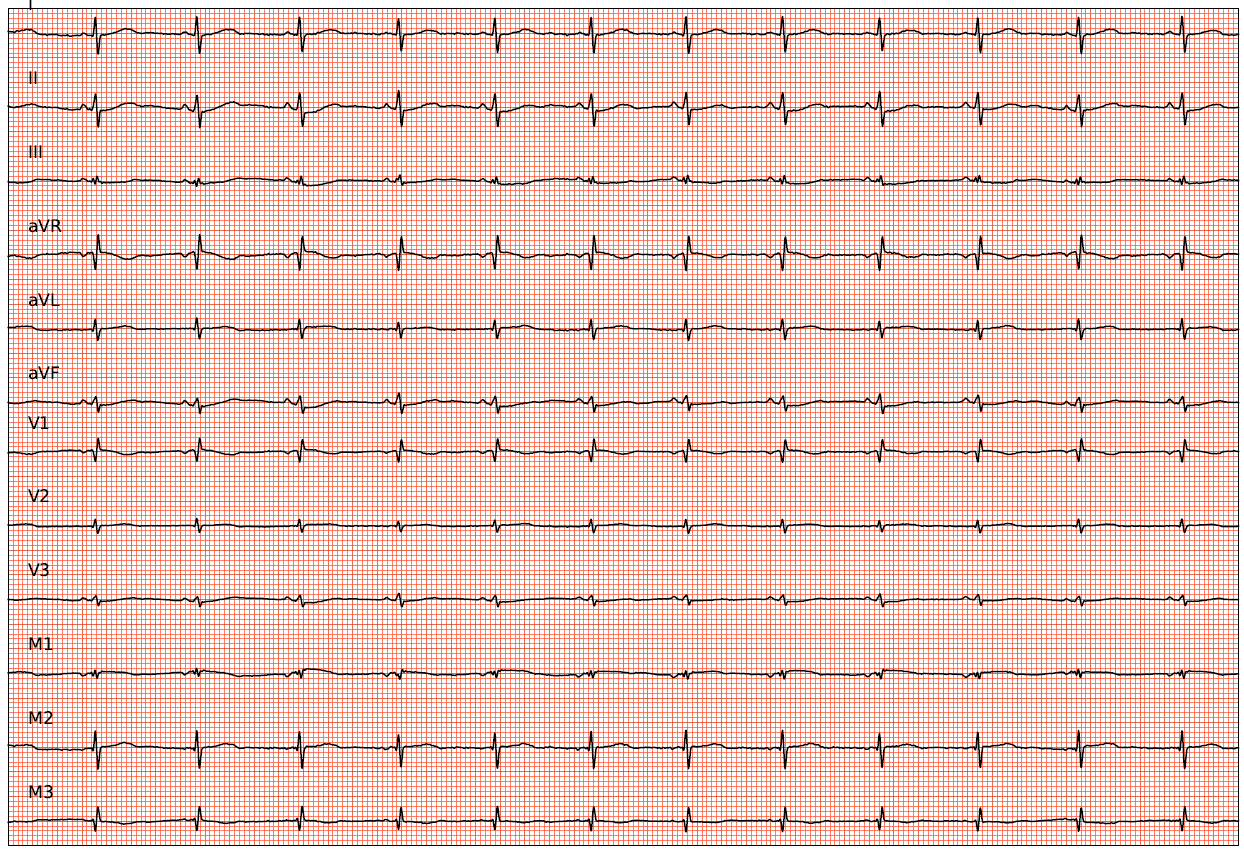


**Supplementary 3 Figure 1.** Normal recording without any noise


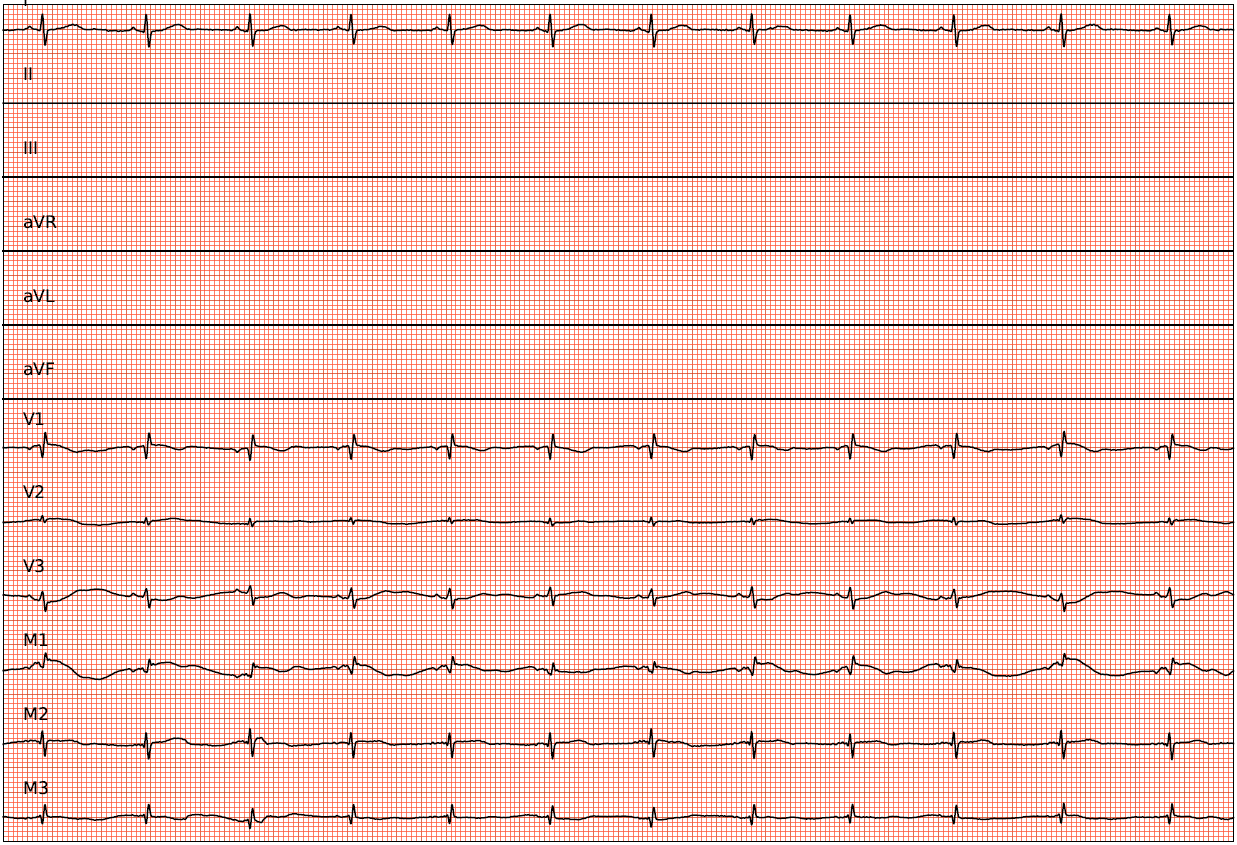


**Supplementary 3 Figure 2.** Recording affected by mechanical strain as screws used for connecting electronic board and electrode were too tight affecting recording in channels II, III and augmented leads.


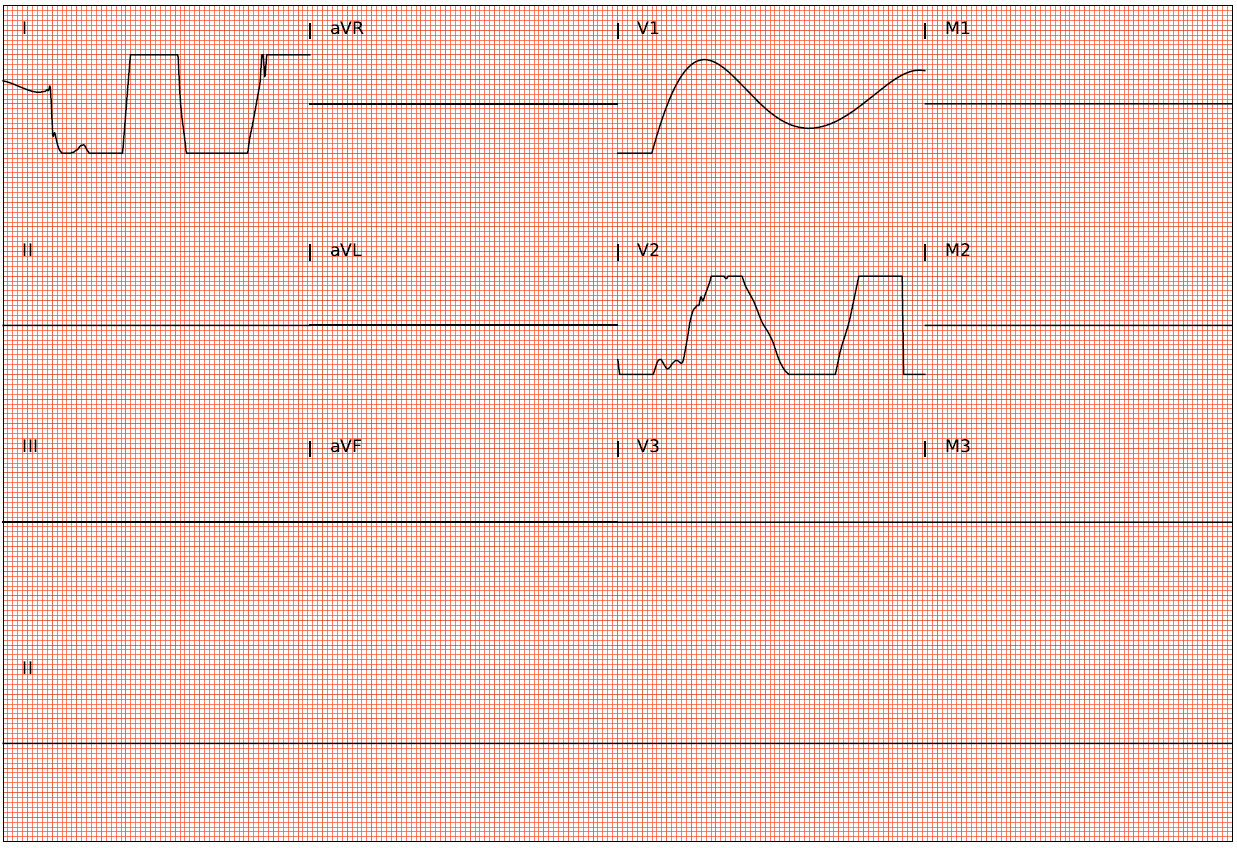


**Supplementary 3 Figure 3.** Recording affected as electrode spray leak into device showing that no channel can record ECG changes.

**
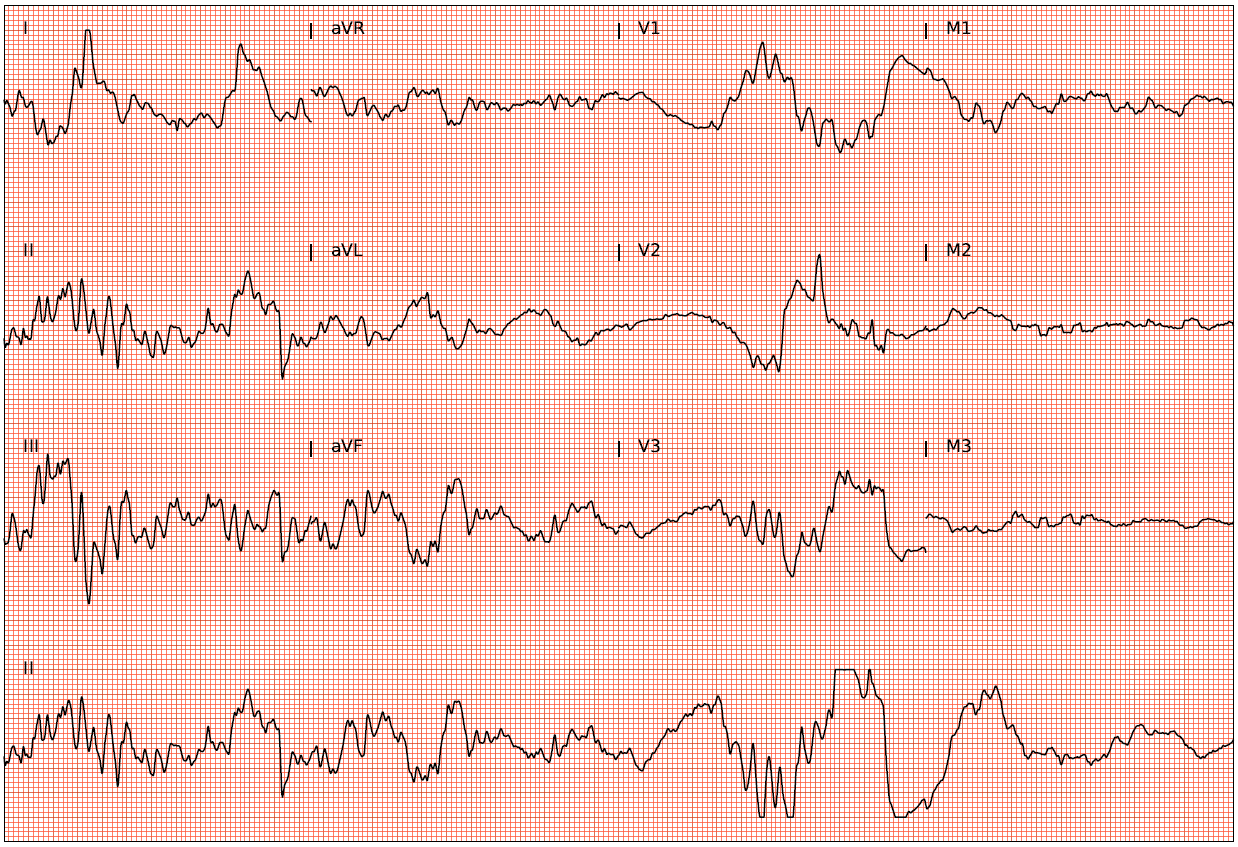
**

**Supplementary 3 Figure 4.** Recording affected as skin-electrode impedance too high for recording.
